# Case report: Increased troponin level in 125 children during COVID-19

**DOI:** 10.3389/fped.2023.1059685

**Published:** 2023-03-01

**Authors:** Paola Di Filippo, Daniela David, Marina Attanasi, Nadia Rossi, Francesco Chiarelli

**Affiliations:** Department of Pediatrics, University of Chieti, Chieti, Italy

**Keywords:** MIS-C, SARS-CoV-2, cardiac involvement, children, troponin

## Abstract

**Introduction:**

Increase in cardiac biomarkers during Coronavirus disease 2019 (COVID-19) was frequent regardless of the presence of myocarditis and multisystem inflammatory syndrome in children (MIS-C). Several studies described MIS-C, but few papers evaluated cardiac manifestations in children with SARS-CoV-2 infection without MIS-C and investigated the role of troponin in absence of electrocardiogram (ECG) and echocardiographic alterations. The aim of this case series is to describe the cardiac manifestations during COVID-19 in children, trying to explain the meaning of laboratory findings during COVID-19, especially of increased troponin.

**Materials and methods:**

We conducted a retrospective case series of children aged <18 years admitted at the Department of Pediatrics, University of Chieti, for SARS-CoV-2 infection between 1st March 2020 and 31th July 2022. All patients with documented SARS-CoV-2 infection underwent a laboratory evaluation at admission. Children with increased troponin I and/or BNP underwent electrocardiographic and echocardiographic exams.

**Results:**

125 children were admitted for SARS-CoV-2 infection to our Department of Pediatrics, of whom 17 (13.6% of cases) with different patterns of cardiac involvement. Specifically, 5 subjects (4.0% of admitted children) were diagnosed as MIS-C and 12 children (9.6%) manifested a cardiac involvement in terms of increased troponin with or without ECG and echocardiography anomalies. Troponin, C-reactive protein, procalcitonin and BNP values resulted higher in patients with MIS-C compared to patients without MIS-C. Furthermore, patients with MIS-C had higher neutrophils and lower lymphocytes compared to patients without MIS-C. ECG abnormalities were found in 4/5 patients with MIS-C and in 2/12 patients without MIS-C. Echocardiographic anomalies were found in all patients with MIS-C, especially in terms of valve regurgitation and ejection fraction reduction and in 2/12 patients without MIS-C, especially in terms of pericardial effusion. Despite high troponin levels, children presented a favorable clinical evolution.

**Conclusion:**

The increase in troponin level in children with COVID-19 could also be due to respiratory causes or a massive inflammatory state. In our case series, patients with increased troponin associated to COVID-19 presented a favorable clinical course with clinical and laboratory remission almost always within 7 days.

## 1. Introduction

At the onset of the Coronavirus disease 2019 (COVID-19) outbreak, children were marginally involved, accounting for 1.7% of cases, and considered mostly asymptomatic carrier cases (1). During the following epidemic waves, an increasing number of children exposed to COVID-19 developed a multisystem inflammatory syndrome in children (MIS-C), defined on May 14, 2020, by the Centers for Disease Control (CDC) with a Health Alert Network (1, 2). Although COVID-19 mostly showed a favorable prognosis in children, MIS-C was characterized by an overwhelming inflammatory activation, with clinical similarities with Kawasaki disease (KD) including cardiac involvement (3).

SARS-CoV-2 mainly affects the upper respiratory tract. Nevertheless, the virus can damage other tissue than lung through a direct injury or an indirect one caused by the release of proinflammatory cytokines (4). An exaggerated inflammatory response triggered by the cytokine storm could cause a multi-organ involvement (5). A higher risk of severity and mortality was described in patients with underlying cardiovascular morbidity (6).

The incidence of MIS-C is not clear, but some estimates showed that MIS-C occurs in 5.1 out of 1,000,000 person-months in individuals under the age of 21 years (7). Cardiac involvement was frequently described in children with MIS-C, with myo-pericardial inflammation, coronary dilatation or aneurysm and arrhythmias (8). The mechanism of myocardial dysfunction in MIS-C is still unclear, but possible causes include acute myocarditis, post-viral immunological reaction, and systemic inflammatory response syndrome (2). The pathophysiology of MIS-C includes a sequence of events. Firstly, neutrophils infiltrate vessel walls causing acute necrotizing arteritis and an aneurysm within the coronary artery. Macrophages and T-cell lymphocytes accumulate in the damaged vessel wall, initiating a chronic form of vasculitis, with proliferation of myofibroblasts and risk for coronary artery stenosis. In the early acute phase, myocardial edema characterizes myocarditis before evidence of an aneurysm, with possibly transient left ventricular dysfunction that can lead to cardiovascular shock (4).

In literature, clinical and laboratory data mostly derived from clinical cases. In a case series of 20 critically ill children admitted for shock, fever and SARS-CoV-2 infection between 15th and 27th April 2020, the authors found an acute myocarditis with a mean left ventricular ejection fraction of 35% and high troponin level (269 ng/ml). The first symptoms before admission were intense abdominal pain and fever for 6 days and all children showed increased inflammation indexes (9).

In a French prospective study including 21 children and adolescents with features of KD admitted between 27 April and 11 May 2020, Toubiana et al. (10) found evidence of recent SARS-CoV-2 infection in 19 (90%) subjects. The authors observed myocarditis in 16 (76%) patients, with a median left ventricular ejection fraction rate of 42%, increased troponin I and B-type natriuretic peptide in 81% and 78% patients respectively, and pericardial effusion in 48% of enrolled children. Interestingly, all 21 patients presented noticeable gastrointestinal symptoms and high levels of inflammatory markers during the early stage of disease.

Furthermore, in a multicenter case series of 183 children with MIS-C, a wide clinical spectrum was found. All patients presented with fever, 63.9% gastrointestinal symptoms, and 43.2% presented with shock. Inotropic support, mechanical ventilation, and extracorporeal membrane oxygenation were indicated in 39.3%, 23.5%, and 2.2% patients, respectively (11).

An increased volume, a diastolic dysfunction and a reduced ejection fraction of the left ventricle were frequently found during echocardiographic exam. On imaging examinations, Magnetic Resonance Imaging (MRI) findings often showed late gadolinium enhancement, native T1 and T2 enhancement, and pericardial enhancement (9). These data suggest that SARS-CoV-2 can cause myocarditis, myocardial ischemia, and heart failure in a significant percentage of infected patients (12).

Children with COVID-19 myocarditis showed higher C-reactive protein levels, variable clinical features, need for shorter inotropic therapy and faster recovery of the left ventricular systolic function compared to patients with non-COVID-19 myocarditis (13).

The management of acute MIS-C patient includes cardiac support, immunomodulation, and antiplatelet/anticoagulant treatments (14). Whittaker et al. (15) documented cardiac involvement with left ventricular dysfunction on echocardiography and troponin elevation in 62% and 66% of children with MIS-C, respectively.

The aim of this case series is to describe the cardiac manifestations during COVID-19 in children, trying to explain the meaning of laboratory findings during COVID-19, especially of increased troponin.

## 2. Materials and methods

We conducted a retrospective case series of all children aged <18 years admitted at the Department of Pediatrics, University of Chieti, for SARS-CoV-2 infection between 1st March 2020 and 31th July 2022. Written informed consent was obtained from the minor's legal guardian for the publication of any potentially identifiable images or data included in this article. We reviewed the medical records of children who needed hospitalization to collect clinical, laboratory, imaging and echocardiographic findings and data about COVID-19 vaccination history. The time between onset of symptoms and admission and days of hospitalization were also recorded.

SARS-CoV-2 was diagnosed at the admission using reverse-transcriptase polymerase chain reaction (PCR) on nasopharyngeal and/or oropharyngeal swab samples. Serological test for SARS-CoV-2 antibodies detection was performed in children without active infection.

Clinical evaluation included physical examination and vital signs. Main clinical symptoms at onset, including fever, mucocutaneous involvement, presence of nonsuppurative laterocervical lymphadenopathy, conjunctivitis, and gastrointestinal, respiratory, cardiovascular and neurologic symptoms were also collected.

All patients with documented SARS-CoV-2 infection underwent a laboratory evaluation at admission, including blood cell count with white blood cells (WBC, 10^3^/mmc), platelet count (PLT, 10^3^/mmc) and hemoglobin (Hb, g/dl); C-reactive protein (CRP, mg/L) and procalcitonin (PCT, mg/ml); troponin I (pg/ml) and brain natriuretic peptid (BNP, pg/ml), transaminases (U/L), ferritin (ng/ml), D-dimer (ng/ml). According to our Laboratory Unit, the upper limit of serum troponin level is 15.2 pg/ml and of BNP is 100 pg/ml.

Children with increased troponin I and/or BNP underwent electrocardiographic and echocardiographic exams. Electrocardiogram (ECG) data, including abnormal PR and QT intervals and ST- and T-wave changes, were recorded. Echocardiography findings were considered, including left ventricular ejection fraction, assessment of coronary arteries, and pericardial effusion.

Cardiac involvement is defined according to World Health Organization (WHO) definition as the presence of myocardial dysfunction, pericarditis, valvulitis, or coronary abnormalities (including findings on echocardiogram or elevated levels of troponin/BNP) (16). Patients with cardiac involvement were included in the analysis and divided into two groups: SARS-CoV-2 patients with and without MIS-C. According to WHO criteria, MIS-C was diagnosed in children and adolescents 0–19 years of age with fever >3 days with evidence of SARS-CoV-2 infection and exclusion of other obvious microbial cause and increased inflammation markers and two the following criteria: (a) Rash or bilateral non-purulent conjunctivitis or muco-cutaneous inflammation signs (oral, hands or feet); (b) Hypotension or shock; (c) cardiac involvement; (d) Evidence of coagulopathy (by prothrombin time, partial thromboplastin time, elevated d-Dimers); (e) Acute gastrointestinal problems (diarrhea, vomiting, or abdominal pain) (16).

In children with MIS-C, usual etiologic causes of acute myocarditis were screened, including testing of a large panel of non-SARS-CoV-2 viruses in blood, as Epstein–Barr virus, Cytomegalovirus, Parvovirus B19, Coxsackievirus, Echovirus. Other causes of infection were also excluded testing culture of urine, stool, and nasopharyngeal swabs and detection of Mycoplasma, Adenovirus, Influenza and Parainfluenza virus antibodies.

Information about need for oxygen, intravenous immunoglobulins, antiplatelet/anticoagulant treatments and inotropic support was collected. The clinical evolution including admission to Intensive Care Unit or death was also considered.

Continuous data was expressed as median and range 5%–95%. Categorical data was presented as numbers and percentages. Mann-Whitney *U* test was performed to compare characteristics between two groups. The statistical significance level was *p* < 0.05. SPSS version 25.0 for Windows (IBM, Armonk, NY, USA) and STATA/IC 15.1 (StataCorp. 2017. Stata *Statistical Software: Release 15*. StataCorp LLC. College Station, TX, USA) were used to perform statistical analysis.

## 3. Results

**Figure 1:**
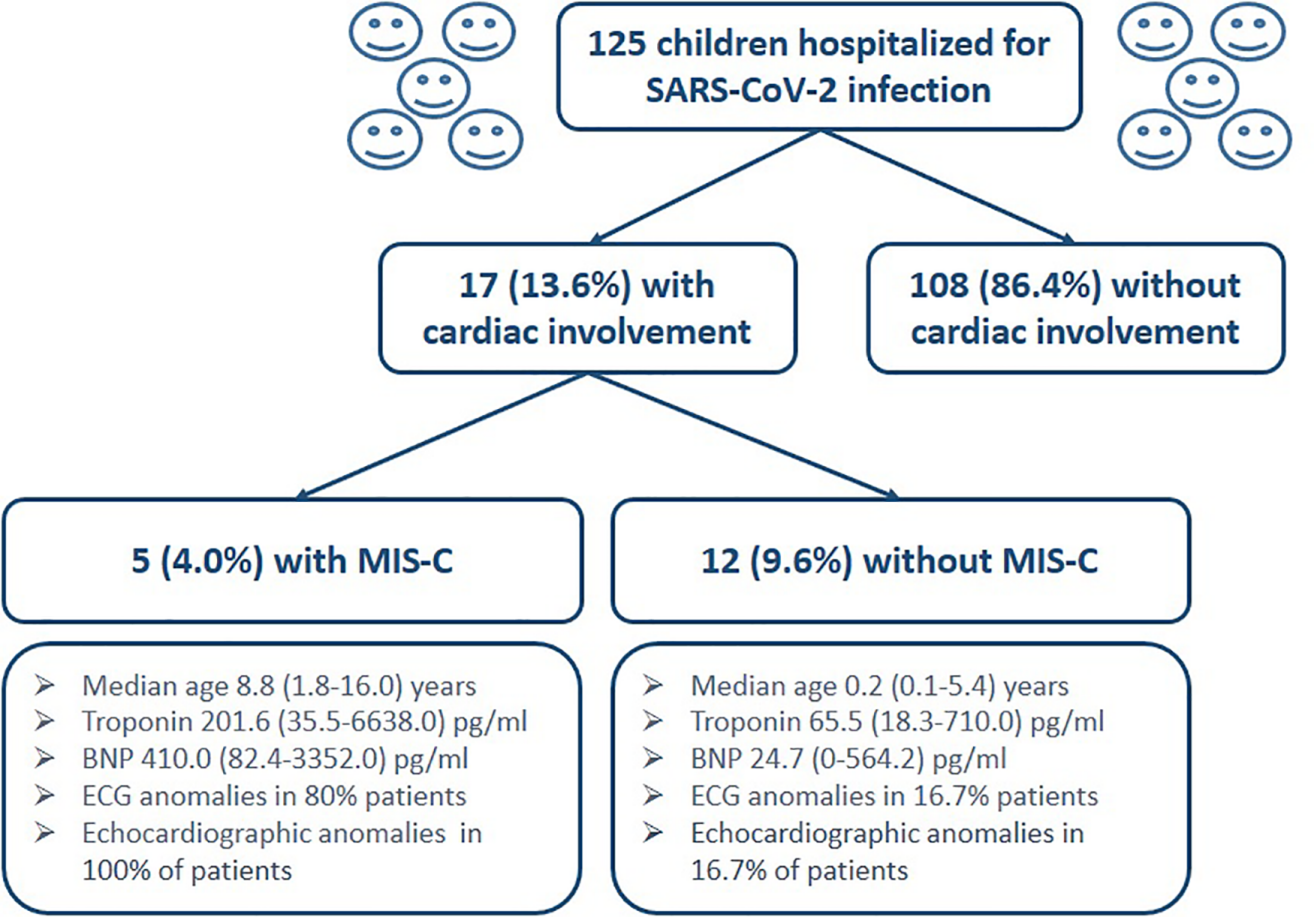
Flow-chart of children enrollment with main laboratory, electrocardiographic and echocardiographic findings in the two groups.

One hundred twenty five children were admitted for SARS-CoV-2 infection to our Department of Pediatrics. Seventeen children (13.6% of cases) showed different patterns of cardiac involvement. Specifically, 5 subjects (4.0% of admitted children) were diagnosed as MIS-C with a median age of 8.8 years (5%–95% CI 1.8–16.0); three were male (60.0%). Twelve children (9.6% of total cases) with a median age of 0.2 years (5%–95% CI 0.1–5.4, male 50%) manifested a cardiac involvement in terms of increased troponin with or without ECG and echocardiography anomalies, and showing no diagnostic criteria for MIS-C (Figure 1). Other etiologic causes of acute myocarditis (Epstein–Barr virus, Cytomegalovirus, Parvovirus B19, Coxsackievirus, Echovirus) resulted negative in children with MIS-C.

Children with MIS-C were older compared to children without MIS-C (8.8 vs. 0.2 years, *p* < 0.001). Patients without MIS-C were mainly infants (11/12 patients, 91.6% of cases), mostly within the first 3 months of life. The child with the highest troponin level (710 picogram/ml) was a 2-month-old infant with a minimal pericardial effusion detected by echocardiography that did not require any intervention. In this case, troponin level gradually decreased fluctuating after approximately 70 days, while increased troponin values in other patients became normal after about a week.

Nine patients (9/108, 8.3%) among children without cardiac involvement and one patient (1/17, 5.9%) with increased troponin levels were previously vaccinated for SARS-CoV-2.

Children with MIS-C presented fever (100% of cases), rash (80.0%), diarrhea (80.0%), conjunctivitis (60.0%), dyspnea (40.0%), mucositis (40.0%) and neurologic symptoms (lethargy, headache, confusion in 40.0% of patients). In patients without MIS-C, clinical characteristics ranged from fever (66.7% of patients), tachypnea (50.0%), cough (41.7%), diarrhea and weight loss (8.3%), headache (8.3%) and tachycardia (8.3%). Patients with MIS-C showed higher troponin levels compared to patients without MIS-C [201.6 (35.5–6638.0) vs. 65.5 (18.3–710.0)] but the difference was not statistically significant (*p* = 0.328). C-reactive protein [158.6 (89.7–109.4) vs. 3.5 (0.2–38.5); *p* < 0.0001], procalcitonin [4.1 (0.04–55.3) vs. 0.2 (0.01–1.4); *p* = 0.048], and BNP [410.0 (82.4–3352.0) vs. 24.7 (0–564.2); *p* = 0.008] values were higher in patients with MIS-C compared to patients without MIS-C. Values of WBS are similar between the two groups [12.8 (5.4–18.3) vs. 9.7 (2.9–19.5); *p* = 0.442], but patients with MIS-C had higher neutrophils [10.7 (CI 4.1–15.5) vs. 2.1 (0.6–7.1); *p* = 0.002] and lower lymphocytes [1.4 (0.7–2.2) vs. 6.7 (1.7–10.9); *p* = 0.002] compared to patients without MIS-C.

Aspartate aminotransferase [28.0 (20.0–156.0) vs. 49.0 (25.0–574.0); *p* = 0.377], alanine aminotransferase [21.0 (19.0–350.0) vs. 43.0 (21.0–406.0); *p* = 0.377], ferritin [378.0 (283.8–524.8) vs. 784.5 (186.4–1755.0); *p* = 0.247] and D-dimer [1.2 (0.5–10.2) vs. 0.9 (0.6–1.5); *p* = 0.524] were not significantly different between the two groups.

ECG abnormalities were found in 4/5 patients (80.0%) with MIS-C (Table 1), while in patients with increased troponin and without MIS-C 2/12 patients (16.7%) showed ECG abnormalities: a patient with ventricular repolarization (VR) abnormalities and one with sinus tachycardia (Table 2).

**Table 1:** Clinical, laboratory and cardiac findings and oxygen need of 5 patients with MIS-C.

Patient	1	2	3	4	5
Age (years)	12.5	6.7	1.8	8.8	16.0
PCR for SARS-CoV-2 on naso/oro-pharyngeal swab	Negative	Negative	Negative	Negative	Negative
Serological test for SARS-CoV-2	Positive	Positive	Positive	Positive	Positive
Clinical characteristics	Fever, rash, dyspnea, diarrhea	Fever, rash, dyspnea, diarrhea	Fever, rash, diarrhea, conjunctivitis, mucositis, lethargy	Fever, conjunctivitis, mucositis, headache, confusion	Fever, rash, rhinitis, diarrhea
Weeks after the positive SARS-CoV-2 swab	4	3	4	4	4
Days between onset of symptoms and admission	4	2	6	7	7
Days of hospitalization	17	12	1 before PICU	12	16
WBC (x 10^3^/µl)	5.4	9.2	18.3	14.4	12.8
N Ratio (%)	76.6	84.3	85.0	75.7	83.8
CRP (mg/L) Nv < 5	166.3	89.7	308.4	158.6	98.0
PCT (ng/ml) Nv < 0.5	4.1	8.9	55.3	1.3	0.04
Troponin (pg/ml) Nv 0.0–15.2	211.9	201.6	35.5	56.7	6638.0
BNP (pg/ml) Nv < 100	240.8	2926.0	3352.0	410.0	88.4
Echocardiographic findings	Mild to moderate mitral regurgitation, EF 55%	Mild tricuspid regurgitation, moderate mitral regurgitation, EF 60%	Left ventricular function at lower limit of normal, EF 50%	Slight reduction in EF (50%)	Minimal mitral and tricuspid regurgitation, EF 55%
ECG	Bradycardia First degree atrioventricular block	Low atrial ectopic rhythm QT interval prolongation	Normal	Sinus bradycardia	Incomplete right bundle branch block
Oxygen need	No	No	No	No	No

Children with MIS-C were older compared to children without MIS-C (8.8 vs. 0.2 years, *p* < 0.001). All patients had a negative swab and positive serology for SARS-CoV-2; the infection dated back to 3–4 weeks ago in all 5 cases. Regarding clinical findings, children with MIS-C presented fever (100% of cases), rash (80.0%), diarrhea (80.0%), conjunctivitis (60.0%), dyspnea (40.0%), mucositis (40.0%) and neurologic symptoms (lethargy, headache, confusion in 40.0% of patients). Regarding cardiac findings in patients with MIS-C, troponin levels resulted increased in all patients and ranged from 35.5 to 6638.0 pg/ml; BNP levels resulted increased in 4 patients (80.0%) and ranged from 82.4 to 3352.0 pg/ml; echocardiographic anomalies were found in all patients, especially in terms of valve regurgitation and ejection fraction reduction; ECG abnormalities were found in 4 patients (80.0%). Furthermore, all patients presented neutrophilia and inflammation indices were increased in almost all patients with MIS-C: CRP was increased in all patients and PCT in 4 patients (80.0%). WBC, white blood cells; N, neutrophil; Hb, hemoglobin; PLTs, platelets; CRP, C-reactive protein; Nv, normal values; PCT, procalcitonin; BNP, brain natriuretic peptid; EF, ejection fraction; MRI, magnetic resonance imaging; ECG, electrocardiogram; O_2_, oxygen.

**Table 2:** Clinical, laboratory and cardiac findings and oxygen need of 12 patients with increased troponin levels without MIS-C.

Patient	1	2	3	4	5	6	7	8	9	10	11	12
Age (years)	0.2	5.4	0.1	0.3	0.2	0.1	0.1	0.2	0.2	0.7	0.2	0.2
PCR for SARS-CoV-2 on naso/oro-pharyngeal swab	Positive	Positive	Positive	Positive	Positive	Positive	Positive	Positive	Positive	Positive	Positive	Positive
Clinical characteristics	Cough Tachypnea	Haedache Tachycardia	Fever	Fever Cough	Fever Tachypnea	Fever Cough Tachypnea	Fever Diarrhea Weight loss	Cough Tachypnea	Fever Tachypnea Perioral cianosis	Fever Cough Tachypnea	Irritability	Fever
Days between onset of symptoms and admission	3	7	1	5	4	1	2	8	2	4	1	3
Days of hospitalization	6	10	5	6	3 before PICU	4	4	24	3	9	16	3
WBC (x 10^3^/µl)	7.09	8.00	7.39	8.41	12.19	2.93	13.02	8.86	14.35	11.13	15.62	6.99
N Ratio (%)	61.7	41.6	19.8	12.4	11.2	22.2	16.8	14.0	15.0	63.8	27.9	33.6
CRP (mg/L) Nv < 5	0.36	20.31	0.96	0.11	2.52	6.68	1.89	0.17	4.53	38.50	0.91	6.77
PCT (ng/ml) Nv < 0.5	n.a.	0.26	0.17	0.07	0.08	0.71	0.46	0.04	1.00	0.13	0.18	0.16
Troponin (pg/ml) Nv 0.0–15.2	96.1	137.7	54.9	18.3	53.6	142.4	62.0	710.0	20.9	196.0	67.0	36.2
BNP (pg/ml) Nv < 100	n.a.	<10	19.1	<10	13.9	30.2	11.3	52.2	n.a.	564.2	82	91.5
Echocardiographic findings	Normal	Normal	Normal	Mild pericardial effusion	Normal	Normal	Normal	Pericardial effusion	Normal	Normal (past surgery for CAVC type C)	Normal	n.a.
ECG	Normal	Sinus tachycardia	Normal	Normal	Normal	Normal	Normal	Normal	Normal	Repolarization abnormalities	Normal	Normal
Oxygen need	No	No	No	No	No	No	No	No	No	Low-flow O2 for 1 day	No	No

Patients without MIS-C were mainly infants (11/12 patients, 91.6% of cases), mostly within the first 3 months of life. All patients had a positive swab for SARS-CoV-2. Clinical findings in patients without MIS-C ranged from fever (66.7% of patients), tachypnea (50.0%), cough (41.7%), diarrhea and weight loss (8.3%), headache (8.3%) and tachycardia (8.3%). Regarding cardiac findings in patients with increased troponin levels and without MIS-C, troponin levels ranged from 18.3 to 710.0 pg/ml. The child with the highest troponin was a 2-month-old infant with a minimal pericardial effusion detected by echocardiography that did not require therapy. In this case, troponin level gradually decreased fluctuating after approximately 70 days, while increased troponin values in other patients became normal after about a week. BNP levels resulted increased in 1 patient (8.3%) and ranged from 0 to 564.2 pg/ml; echocardiographic anomalies were found in 2/12 patients (16.7%), especially in terms of pericardial effusion; ECG abnormalities were found in 2/12 patients (16.7%), a patient with ventricular repolarization abnormalities and one with sinus tachycardia. Furthermore, neutrophilia was less frequent compared to subjects with MIS-C, as well as inflammation indices were less frequently and deeply elevated: CRP was increased in 4 patients (33.3%) and PCT in only one patient (8.3%). WBC, white blood cells; N, neutrophil; Hb, hemoglobin; PLTs, platelets; CRP, C-reactive protein; Nv, normal values; PCT, procalcitonin; BNP, brain natriuretic peptid; ECG, electrocardiogram; CAVC, complete atrioventricular canal; O_2_, oxygen.

Echocardiographic anomalies were found in all patients with MIS-C, especially in terms of valve regurgitation and ejection fraction reduction (Table 1). Conversely, echocardiographic anomalies were found in 2/12 patients (16.7%), especially in terms of pericardial effusion (Table 2).

One patient (20.0%) in the MIS-C group and one patient (8.3%) in the group without MIS-C were transferred to the Pediatric Intensive Care Unit. None of the 17 patients presented sequelae. None of the 17 patients died, but a patient in the whole group of infected children (1/125, 0.8%) died because of respiratory failure. The patient had Down syndrome and congenital heart disease (subaortic ventricular defect and patent foramen ovale with pulmonary hypertension, previously surgically corrected).

## 4. Discussion

In this case series, cardiac involvement was detected in 13.6% (17/125) and MIS-C in 4.0% (5/125) of hospitalized children with a recent history of COVID-19 or acute COVID-19. All the patients with MIS-C showed increased troponin level, while 12 children (9.6% patients) with current SARS-CoV-2 infection presented increased troponin levels. Despite high troponin level, only 2/12 patients had ECG anomalies and 3/12 echocardiographic ones.

In literature, several studies described MIS-C and the role of troponin increase as a marker of myocardial damage was widely reported (17–19). Otherwise, few papers evaluated cardiac manifestations in children with SARS-CoV-2 infection without MIS-C and investigated the role of troponin in absence of ECG and echocardiographic alterations. Noteworthy, increase in cardiac biomarkers during COVID-19 was frequent regardless of the presence of myocarditis, especially in the phase of severe systemic inflammation and acute respiratory distress syndrome and quantitatively associated with poor outcome (20). Indeed, an increase in cardiac biomarkers was found in up to 27% of COVID-19 patients (21).

In a single-center retrospective observational study of 759 children with increased troponin levels over an 11-year period, the authors found that this laboratory finding was associated mostly to cardiac diseases. Nevertheless, increased troponin resulted also in drug or carbon monoxide intoxication, bronchopneumonia, asthma, sepsis, septic shock, and systemic inflammatory response syndrome and hypotension or hypovolemia (22). Therefore, Yoldas et al. (22) showed that troponin elevation could be caused also by non-cardiac disease.

We speculated that the frequent increased troponin levels in children with COVID-19 could not be due to viral infection of the heart. Indeed, in a study including COVID-19 patients increased troponin levels were not correlated with left ventricular dysfunction, but with right ventricular one. These findings suggested that increased troponin was due to acute right ventricular overload secondary to parenchymal or vascular lung disease resulting in subendocardial damage of the right ventricular myocardium (23). However, respiratory distress was not significant in our 12 patients with troponin increase, such that only one required oxygen. Therefore, in children infected and without myocarditis, the increase in troponin could be mostly related to the intense inflammatory state (22).

Otherwise, another hypothesis could be that very mild myocarditis, not evident on ECG and echocardiogram findings, resulted only in troponin increase. Tunçer et al. (24) reported that a mild myocarditis evolved with complete recovery without complications, albeit elevated troponin levels. ECG anomalies were detected in 93%–100% of children with myocarditis in retrospective studies; therefore, a myocarditis without ECG alterations was described rarely (25–27). We speculated that mild cardiac involvement with no clinical significance could be detected only by laboratory tests.

Comparing patients with and without MIS-C, we found higher troponin level in patients with MIS-C, although not significantly. Furthermore, we found significant higher levels of neutrophils, C-reactive protein, procalcitonin and BNP and significant lower values of lymphocytes in patients with MIS-C compared to patients without MIS-C. Similarly, in a recent study with 233 children with MIS-C and 102 with COVID-19, patients with MIS-C had significant higher levels of troponin, BNP and C-reactive protein and lower lymphocytes compared with COVID-19 children (28). We suggested that these laboratory findings could help the physician to differentiate patients with MIS-C. Particularly, BNP seems to be a promising severity index. In a recent metanalysis including 24 studies comprised of 2,583 COVID-19 patients and 1,613 MIS-C patients, the authors suggested that BNP was the key cardiac marker that showed differences between patients with MIS-C/non-severe COVID-19 and between patients with severe/non-severe MIS-C. The authors found that other markers, such as troponin and transaminases, did not exhibit notable differences in indicating cardiac injury between patients with MIS-C and COVID-19 (29).

In our study population, ECG abnormalities were found in 80% of patients with MIS-C and 16.7% of patients without MIS-C. In literature, arrhythmic manifestations were described in a wide range of patients (from 7% to 60%) with MIS-C (14). The most frequently described ECG anomalies were non-specific anomalies of ventricular repolarization, prolongation of the QT interval and premature atrial or ventricular beats (14). First and second degree atrioventricular (AV) blocks and atrial fibrillation were also reported (8, 30). In literature, less information were reported regarding ECG changes in children with troponin elevation without MIS-C. In the 12 children without MIS-C, we found no ECG changes that were clinically significant: one reported only sinus tachycardia and the other ventricular repolarization. Furthermore, the latter was a child with a history of previous surgery for complete atrioventricular canal.

Echocardiography has a key role for the diagnosis and monitoring of myocarditis (31). In our study population, echocardiographic anomalies were found in all patients with MIS-C (especially in terms of valve regurgitation and ejection fraction reduction), confirming the key role of this tool in the diagnosis of MIS-C. Echocardiographic anomalies were also found in 16.7% of patients without MIS-C, especially in terms of pericardial effusion, probably indicating very mild pericarditis. In literature, cardiac complications occurred in approximately 30% of infected children and in nearly all those with MIS-C (2). In a study including 294 children with active or previous SARS-CoV-2 infection, of which 46 with MIS-C, the most frequent echocardiographic change was pericardial effusion, followed by left ventricular systolic dysfunction, while coronary abnormalities occurred in about one third of patients with MIS-C (2).

The lack of MRI data in patients with cardiac involvement is a limitation in this case series. Indeed, cardiac MRI is the gold standard for the quantitative evaluation of ventricular systolic function and it could detect myocardial edema. Verification of myocarditis in patients with acute cardiac syndromes but normal coronary arteries or with atypical symptoms is one of its greatest challenges in clinical practice (32).

Regarding clinical evolution, in our population one patient in the MIS-C group and one patient in the group without MIS-C were transferred to a Pediatric Intensive Care Unit. However, the clinical evolution was favorable for all patients with complete clinical remission; none of the 17 patients presented sequelae or died. In literature, a mortality rate of 1% in children with MIS-C after adequate treatment was reported (33). In a large meta-analysis of 42 studies including 275,661 children without comorbidities and 9,353 children with comorbidities, a mortality rate of 0.03% in children without comorbidities and of 1.5% in children with pre-existing comorbidities was found (34). In our study population, a patient in the whole group of infected children (1/125, 0.8%) died because of respiratory failure. The patient had Down syndrome and congenital heart disease (subaortic ventricular defect and patent foramen ovale with pulmonary hypertension, previously surgically corrected).

## 5. Conclusion

We found a 4% prevalence of MIS-C in subjects hospitalized for COVID-19 and a 9.6% prevalence of troponin elevation in patients infected without MIS-C. Additionally, children with MIS-C were older, had higher neutrophils, inflammation indexes, troponin and BNP levels and lower lymphocytes compared to children without MIS-C.

This case series is one of the few investigating the role of troponin in SARS-CoV-2 infected children without MIS-C. The significance of troponin increase not associated to ECG and echocardial anomalies is not yet clear. Surely, it must be kept in mind that the increase in troponin level can also occur in case of right ventricle overload due to respiratory causes or in case of a massive inflammatory state. However, this increase could also be caused by a very slight myocardial damage, not detectable on ECG and echocardiogram. In our case series, patients with increased troponin associated to SARS-CoV-2 infection presented a favorable clinical course with clinical and laboratory remission almost always within 7 days.

Nevertheless, follow-up of children with cardiac involvement associated to SARS-CoV-2 infection remains to be clarified. It is not clear whether a control echocardiogram is necessary in all patients with increased troponin. Larger multicenter studies to better define the incidence and characteristics of cardiac involvement associated to SARS-CoV-2 infection in children are needed. Finally, being a novel entity, long-term studies are needed to better define evolution and prognosis of this disease in children.

## Data Availability

The original contributions presented in the study are included in the article/Supplementary Material, further inquiries can be directed to the corresponding author.

## References

[1] CDC COVID-19 Response Team. Coronavirus disease 2019 in children - United States, February 12-April 2, 2020. Morb Mortal Wkly Rep. (2020) 69(14):422–6. 10.15585/mmwr.mm6914e4 PMC7147903 32271728

[2] Cantarutti N Battista V Adorisio R Cicenia M Campanello C Listo E Cardiac manifestations in children with SARS-COV-2 infection: 1-year pediatric multicenter experience. Children. (2021) 8(8):717. 10.3390/children8080717 34438608 PMC8392006

[3] Soumya RS Unni TG Raghu KG. Impact of COVID-19 on the cardiovascular system: a review of available reports. Cardiovasc Drugs Ther. (2021) 35(3):411–25. 10.1007/s10557-020-07073-y 32926272 PMC7487338

[4] Abi Nassif T Fakhri G Younis NK Zareef R Al Amin F Bitar F Cardiac manifestations in COVID-19 patients: a focus on the pediatric population. Can J Infect Dis Med Microbiol. (2021) 2021:5518979. 10.1155/2021/5518979 34326911 PMC8287458

[5] van Doremalen N Bushmaker T Morris DH Holbrook MG Gamble A Williamson BN Aerosol and surface stability of SARS-CoV-2 as compared with SARS-CoV-1. N Engl J Med. (2020) 382(16):1564–7. 10.1056/NEJMc2004973 32182409 PMC7121658

[6] Tan W Aboulhosn J. The cardiovascular burden of coronavirus disease 2019 (COVID-19) with a focus on congenital heart disease. Int J Cardiol. (2020) 309:70–7. 10.1016/j.ijcard.2020.03.063 32248966 PMC7102656

[7] Dufort EM Koumans EH Chow EJ Rosenthal EM Muse A Rowlands J Multisystem inflammatory syndrome in children in New York state. N Engl J Med. (2020) 383:347–58. 10.1056/NEJMoa2021756 32598830 PMC7346766

[8] Di Filippo P Raso M Cacciatore M Patacchiola R Renda G Rossi N Case report: mitral valve involvement and first-degree atrial-ventricular block in two patients with multisystem inflammatory syndrome in children. Front Pediatr. (2021) 9:676934. 10.3389/fped.2021.676934 34422717 PMC8377535

[9] Grimaud M Starck J Levy M Marais C Chareyre J Khraiche D Acute myocarditis and multisystem inflammatory emerging disease following SARS-CoV-2 infection in critically ill children. Ann Intensive Care. (2020) 10(1):69. 10.1186/s13613-020-00690-8 32488505 PMC7266128

[10] Toubiana J Poirault C Corsia A Bajolle F Fourgeaud J Angoulvant F Kawasaki-like multisystem inflammatory syndrome in children during the COVID-19 pandemic in Paris, France: prospective observational study. Br Med J. (2020) 369:m2094. 10.1136/bmj.m2094 32493739 PMC7500538

[11] Bautista-Rodriguez C Sanchez-de-Toledo J Clark BC Herberg J Bajolle F Randanne PC Multisystem inflammatory syndrome in children: an international survey. Pediatrics. (2021) 147(2):e2020024554. 10.1542/peds.2020-024554 33234669

[12] Fried JA Ramasubbu K Bhatt R Topkara VK Clerkin KJ Horn E The variety of cardiovascular presentations of COVID-19. Circulation. (2020) 141(23):1930–6. 10.1161/CIRCULATIONAHA.120.047164 32243205 PMC7314498

[13] Vukomanovic VA Krasic S Prijic S Ninic S Minic P Petrovic G Differences between pediatric acute myocarditis related and unrelated to SARS-CoV-2. Pediatr Infect Dis J. (2021) 40(5):e173–8. 10.1097/INF.0000000000003094 33847291

[14] Sperotto F Friedman KG Son MBF VanderPluym CJ Newburger JW Dionne A. Cardiac manifestations in SARS-CoV-2-associated multisystem inflammatory syndrome in children: a comprehensive review and proposed clinical approach. Eur J Pediatr. (2021) 180(2):307–22. 10.1007/s00431-020-03766-6 32803422 PMC7429125

[15] Whittaker E Bamford A Kenny J Kaforou M Jones CE Shah P Clinical characteristics of 58 children with a pediatric inflammatory multisystem syndrome temporally associated with SARS-CoV-2. J Am Med Assoc. (2020) 324(3):259–69. 10.1001/jama.2020.10369 PMC7281356 32511692

[16] World Health Organization. Multisystem inflammatory syndrome in children and adolescents with COVID-19: Scientific brief (2020). Available at: https://www.who.int/publications/i/item/multisystem-inflammatory-syndrome-in-children-and-adolescents-with-covid-19 (Accessed July 25, 2022).

[17] Cui Y Tian M Huang D Wang X Huang Y Fan L A 55-day-old female infant infected with 2019 novel coronavirus disease: presenting with pneumonia, liver injury, and heart damage. J Infect Dis. (2020) 221(11):1775–81. 10.1093/infdis/jiaa113; Erratum in: J Infect Dis. 2020;222(3):519. 32179908 PMC7184483

[18] Li Y Guo F Cao Y Li L Guo Y. Insight into COVID-2019 for pediatricians. Pediatr Pulmonol. (2020) 55(5):E1–4. 10.1002/ppul.24734 32187887 PMC7167677

[19] Su L Ma X Yu H Zhang Z Bian P Han Y The different clinical characteristics of corona virus disease cases between children and their families in China - the character of children with COVID-19. Emerg Microbes Infect. (2020) 9(1):707–13. 10.1080/22221751.2020.1744483 32208917 PMC7103724

[20] Kurz DJ Eberli FR. Cardiovascular aspects of COVID-19. Swiss Med Wkly. (2020) 150:w20417. 10.4414/smw.2020.20417 33382450

[21] Guo T Fan Y Chen M Wu X Zhang L He T Cardiovascular implications of fatal outcomes of patients with coronavirus disease 2019 (COVID-19). JAMA Cardiol. (2020) 5(7):811–8. 10.1001/jamacardio.2020.1017 32219356 PMC7101506

[22] Yoldaş T Örün UA. What is the significance of elevated troponin I in children and adolescents? A diagnostic approach. Pediatr Cardiol. (2019) 40(8):1638–44. 10.1007/s00246-019-02198-w 31485699

[23] Szekely Y Lichter Y Taieb P Banai A Hochstadt A Merdler I Spectrum of cardiac manifestations in COVID-19: a systematic echocardiographic study. Circulation. (2020) 142(4):342–53. 10.1161/CIRCULATIONAHA.120.047971 32469253 PMC7382541

[24] Tunçer T Varol F Coşkun Ş Güzel B Güven Ş Çam H. Patterns of myocardial involvement during COVID-19 pandemic; from newborn to adolescents. J Curr Pediatr. (2021) 19:319–27. 10.4274/jcp.2021.83798

[25] Durani Y Egan M Baffa J Selbst SM Nager AL. Pediatric myocarditis: presenting clinical characteristics. Am J Emerg Med. (2009) 27(8):942–7. 10.1016/j.ajem.2008.07.032 19857412

[26] Freedman SB Haladyn JK Floh A Kirsh JA Taylor G Thull-Freedman J. Pediatric myocarditis: emergency department clinical findings and diagnostic evaluation. Pediatrics. (2007) 120(6):1278–85. 10.1542/peds.2007-1073 18055677

[27] Chang YJ Chao HC Hsia SH Yan DC. Myocarditis presenting as gastritis in children. Pediatr Emerg Care. (2006) 22(6):439–40. 10.1097/01.pec.0000221346.64991.e7 16801847

[28] Godfred-Cato S Abrams JY Balachandran N Jaggi P Jones K Rostad CA Distinguishing multisystem inflammatory syndrome in children from COVID-19, kawasaki disease and toxic shock syndrome. Pediatr Infect Dis J. (2022) 41(4):315–23. 10.1097/INF.0000000000003449 35093995 PMC8919949

[29] Zhao Y Patel J Huang Y Yin L Tang L. Cardiac markers of multisystem inflammatory syndrome in children (MIS-C) in COVID-19 patients: a meta-analysis. Am J Emerg Med. (2021) 49:62–70. 10.1016/j.ajem.2021.05.044 34082189 PMC8129790

[30] Deza Leon MP Redzepi A McGrath E Abdel-Haq N Shawaqfeh A Sethuraman U COVID-19-associated pediatric multisystem inflammatory syndrome. J Pediatric Infect Dis Soc. (2020) 9(3):407–8. 10.1093/jpids/piaa061 32441749 PMC7313914

[31] Dancea AB. Myocarditis in infants and children: a review for the paediatrician. Paediatr Child Health. (2001) 6(8):543–5. 10.1093/pch/6.8.543 20084124 PMC2805590

[32] Friedrich MG Marcotte F. Cardiac magnetic resonance assessment of myocarditis. Circ Cardiovasc Imaging. (2013) 6(5):833–9. 10.1161/CIRCIMAGING.113.000416 24046380

[33] Acevedo L Piñeres-Olave BE Niño-Serna LF Vega LM Gomez IJA Chacón S Mortality and clinical characteristics of multisystem inflammatory syndrome in children (MIS-C) associated with COVID-19 in critically ill patients: an observational multicenter study (MISCO study). BMC Pediatr. (2021) 21(1):516. 10.1186/s12887-021-02974-9 34794410 PMC8600488

[34] Frenkel LD Gomez F Bellanti JA. COVID-19 in children: pathogenesis and current status. Allergy Asthma Proc. (2021) 42(1):8–15. 10.2500/aap.2021.42.200104 33404385

